# Differences Between the 2016 and 2022 Food and Drug Administration Guidance—Implications for Design and Interpretation of Clinical Trials in Ulcerative Colitis

**DOI:** 10.1093/crocol/otae038

**Published:** 2024-06-14

**Authors:** Jurij Hanzel, Christopher Ma, Laurent Peyrin-Biroulet, Silvio Danese, Bruce E Sands, Vipul Jairath

**Affiliations:** Department of Gastroenterology, Faculty of Medicine, University of Ljubljana, Ljubljana, Slovenia; Alimentiv Inc., London, Ontario, Canada; Alimentiv Inc., London, Ontario, Canada; Division of Gastroenterology & Hepatology, Cumming School of Medicine, University of Calgary, Calgary, AL, Canada; Department of Community Health Sciences, Cumming School of Medicine, University of Calgary, Calgary, AL, Canada; INSERM, NGERE, University of Lorraine, Nancy, France; INFINY Institute, Nancy University Hospital, Vandœuvre-lès-Nancy, France; FHU-CURE, Nancy University Hospital, Vandœuvre-lès-Nancy, France; GroupeHospitalier Privé Ambroise Paré – Hartmann, Paris IBD Center, Neuilly sur Seine, France; Gastroenterology and Gastrointestinal Endoscopy Unit, IRCCS San Raffaele Scientific Institute, Vita-Salute San Raffaele University, Milan, Italy; Dr Henry D. Janowitz Division of Gastroenterology, Icahn School of Medicine at Mount Sinai, New York, NY, USA; Alimentiv Inc., London, Ontario, Canada; Department of Medicine, Division of Gastroenterology, University of Western Ontario, London, ON, Canada; Department of Epidemiology and Biostatistics, University of Western Ontario, London, ON, Canada

**Keywords:** clinical trials, sigmoidoscopy, colonoscopy

## Abstract

**Background:**

In 2022, the Food and Drug Administration (FDA) updated its draft guidance for drug development in ulcerative colitis, replacing the version from 2016. Several changes from the 2016 version merit further discussion as they impact clinical trial design and the interpretation of trial results.

**Methods:**

We compared both documents and critically appraised the changes and implications for future clinical trials.

**Results:**

The 2022 guidance recommends full colonoscopy, rather than flexible sigmoidoscopy, to document disease activity in all involved segments of the colon. The concordance between the findings of the 2 procedures is very high and there is little evidence to support colonoscopy over sigmoidoscopy. The use of colonoscopy, rather than sigmoidoscopy, is also associated with a higher burden to trial participants who must undergo full bowel preparation, cost, and a potential for more adverse events. The definition of the Mayo endoscopic score of 0 was changed from the original publication to “normal appearance of mucosa,” which suggests that endoscopic signs of prior disease, such as pseudopolyps and scarring, are incompatible with a score 0, even though they are not associated with active disease. The term “mucosal healing” has been abolished and histologic outcomes defined as exploratory. A welcome change is that shorter washout periods than 5 half-lives will be considered to reduce patient exposure to corticosteroids as bridging therapy.

**Conclusions:**

The 2022 FDA draft guidance includes changes which for the most part are not informed by empirical evidence, which may ultimately complicate interpretation of future trials and preclude comparisons with past trials.

## Teaser

The 2022 draft guidance for drug development in ulcerative colitis by the Food and Drug Administration has several changes compared to the 2016 guidance; notably, it mandates full colonoscopy, rather than sigmoidoscopy.

## Introduction

In 2022, the Food and Drug Administration (FDA) updated its draft guidance for drug development in ulcerative colitis (UC),^1^ replacing the version from 2016,^2^ which was withdrawn and never developed further. Acknowledging that the current document is a non-binding draft and intended as guidance for sponsors, several changes from the 2016 version merit further discussion as they impact clinical trial design and the interpretation of trial results.

## Methods

We compared both documents for key changes and discussed their implications.

## Results

The differences between the 2 draft guidance documents are presented in Table 1.

**Table 1.** Summary of changes between the 2016 and 2022 Food and Drug Administration (FDA) draft guidance for drug development in ulcerative colitis.

	2022 draft guidance	2016 draft guidance	Comments
Method of endoscopic assessment—sigmoidoscopy or colonoscopy?	Colonoscopy recommended to document disease activity in all involved segments of the colon	No specific recommendation for one modality over the other, historically, sigmoidoscopy was used	No evidence to support superiority of colonoscopy over sigmoidoscopy Low rate of discordance between the 2 methods in trial and routine care settings (0.7–5.8%) IBD drug development to date based on sigmoidoscopy Higher cost of colonoscopy Unnecessary burden to trial participants through colonoscopy Potential for more procedure-related adverse events Potential for limiting comparison with past trials Doubtful impact of isolated right-sided inflammation on disease prognosis
Definition of Mayo endoscopic subscore 0	Normal appearance of mucosa	Normal or inactive disease	The 2016 guidance follows the original published index Unclear whether signs of past disease (eg, scarring, atrophy, pseudopolyps) fall under the definition of Mayo endoscopic score of 0 in the 2022 guidance
Definition of clinical remission	Stool frequency subscore = 0 or 1 Rectal bleeding subscore = 0 Centrally read endoscopy subscore = 0 or 1 A stool frequency subscore of 1 is allowable for individual subjects, a subscore of 1 in a significant number of patients may not be considered adequate evidence of stool normalization	Stool frequency subscore = 0 Rectal bleeding subscore = 0 Endoscopy subscore = 0 or 1 Alternative: ≥ 1 point decrease in stool frequency subscore from baseline to achieve 0 or 1. This definition would not support labeling claim of normalization of stool frequency	Appropriate change, approximately 50% of patients with a stool frequency subscore of 1 have endoscopy subscores of 0 or 1
Definition of clinical response	Defined as a decrease from baseline in the modified Mayo score of ≥ 2 points and ≥ 30% reduction from baseline, and a decrease in rectal bleeding subscore of ≥ 1 or an absolute rectal bleeding subscore of 0 or 1	Defined as a decrease in Mayo Score of ≥ 3 points and ≥ 30% reduction from baseline with an accompanying decrease in rectal bleeding subscore ≥1 point or absolute rectal bleeding subscore of 0 or 1	More liberal definition of clinical response with a numerically smaller threshold
Definition of rectal bleeding subscores	1: stool with streaks of blood 2: stool with more than streaks of blood	1: streaks of blood with stool less than half the time 2: obvious blood (more than just streaks) or streaks of blood with stool most of the time The Rectal Bleeding subscore may not be well-defined primarily because it requires patients to report the answer to a double-barreled question (it asks patients to choose streaks of blood with stool less than half the time versus obvious blood with stool most of the time)	Unknown impact of departure from original wording, no empiric evidence of ambiguity of original wording
Corticosteroid-free remission	Defined as patients in clinical remission at the conclusion of the trial and no corticosteroid exposure during a prespecified period (eg, at least 8 to 12 weeks) before that assessment	Definition based on a minimum duration of time over which a patient is both corticosteroid-free and in clinical remission; adequate justification should be provided for the proposed minimum duration	Welcome simplification of definition
Mucosal healing and histologic assessment	There is no consensus on the definition of “mucosal healing,” the FDA does not recommend using it. Histologic endpoints are exploratory, there is no scientific consensus on definitions or scoring systems	To support claims of mucosal healing, endoscopic assessment should be supplemented with validated histological assessment of the mucosa There are limitations to histologic scoring systems and standards of defining histologic improvement Histologic endpoints should be evaluated in phase 2 trials to inform effective incorporation of these assessments in phase 3 trials	The shift in guidance is surprising, given that newly developed drugs have achievement of mucosal healing as a labeling claim Randomized studies to evaluate the incremental benefit of histologic remission beyond endoscopic improvement are ongoing
Washout period	In general, the FDA has recommended a washout of 5 half-lives or undetectable serum concentrations. To promote timely enrollment and reduce corticosteroid bridging therapy, sponsors may propose shorter washout periods with appropriate justification.	No specific recommendation	Welcome change to minimize corticosteroid exposure and to broaden trial participation Small molecules have shorter half-lives and permit shorter washout periods No concerning safety signals from emerging combination trials
Data collection for calculation of stool frequency and rectal bleeding subscores	Daily subscores should be averaged over 7 days (excluding the day of bowel preparation and day of endoscopy). A minimum of 3 consecutive days or 4 nonconsecutive days are necessary	Either the average or the worst of the most recent 3-day consecutive period has been used. Alternatively, at least 3 days (including nonconsecutive days) can be considered.	Minor change, firmer guidance on number of nonconsecutive days

## Discussion

The most notable change is the recommendation for using full colonoscopy, rather than sigmoidoscopy, for endoscopic assessment to document disease activity in all involved segments of the colon. Historically, trials have used sigmoidoscopy and the Mayo endoscopic score (MES),^3^ which continues to be used in contemporary trials with minor modification, was also developed based on sigmoidoscopic assessment.

Regardless, there is limited evidence to support the use of colonoscopy over sigmoidoscopy in this context. In the context of a clinical trial, the agreement between sigmoidoscopy and colonoscopy was assessed in a phase 2 trial of etrolizumab.^4^ The findings of the 2 procedures we highly correlated, both for defining active disease and for remission. Discordant findings at baseline were observed just in 2/100 (2%) of procedures where sigmoidoscopy failed to detect active disease in proximal colonic segments. The rate of disagreement at follow-up examinations was 7/139 (5.0%) for endoscopic improvement (MES ≤ 1) and 1/139 (0.7%) for endoscopic remission (MES 0). In this specific trial, discordances were more common in the placebo group and led to an attenuation of the treatment effect. Spatial evolution of endoscopic healing was also studied in a retrospective cohort study of 86 patients with pancolitis undergoing consecutive colonoscopies—in the absence of centralized reading, these results are not necessarily transferrable to clinical trials.^5^ Nonetheless, the rate of misclassification was low: 3.5% for endoscopic improvement and 5.8% for endoscopic remission.

The clinical relevance of a cecal patch or limited right-sided inflammation in a patient with an otherwise normal colon remains unknown with mainly retrospective and cross-sectional studies yielding conflicting results.^6^ However, it is unlikely that such a finding would contribute to patients’ symptoms or adverse outcomes. The use of colonoscopy, rather than sigmoidoscopy, is also associated with a higher burden to trial participants who must undergo full bowel preparation, cost, and a potential for more adverse events, especially in settings where sedation is used for colonoscopy, but not sigmoidoscopy. Sigmoidoscopy is generally rated by patients as less burdensome than a full colonoscopy.^7^ The shift from sigmoidoscopy to colonoscopy also hinders the comparison of newly developed treatments with all existing approved therapies.

The definition of MES 0 in the modified Mayo score has also been changed. The 2016 draft guidance followed the definition from the original publication, allowing signs of inactive disease, such as pseudopolyps, scarring, and mucosal atrophy. The 2022 document defines a MES 0 as having a “normal appearance of mucosa,” which suggests that the abovementioned endoscopic changes are not compatible with MES 0 as these would not be present on the normal mucosa, never affected by UC. The lack of additional clarification of this change could lead to ambiguity in defining MES 0 and potentially inconsistent centralized scoring between trials, which would further limit comparability between trials. No explanation is provided for this change in the guidance document.

A further change is the threshold of stool frequency to define clinical remission. The 2016 guidance stipulated a stool frequency subscore of 0 and an alternative definition of at least 1-point decrease from baseline to achieve a score of 0/1, whereas the current guidance allows a subscore of 0/1, with a remark that a subscore of 1 in a “significant” number of patients may not be considered sufficient evidence of stool normalization and lead to noting this limitation in the product label. This change is appropriate as approximately 50% of patients with a stool frequency subscore of 1 have endoscopic subscores consistent with clinical remission.^8,9^ The wording for assessing rectal bleeding has also been modified in the 2022 guidance: from “streaks of blood with stool less than half the time” to “stool with streaks of blood” and from “obvious blood with stool most of the time” to “stool with more than streaks of blood,” thereby eliminating the need for patients to assess both the amount of blood and the proportion of bowel movements with admixed blood. No rationale or assessment of the potential impact of this change is provided, but the need for patients to estimate 2 dimensions in a single question is alluded to in the 2016 guidance.

A notable shift from the 2016 guidance is the elimination of the term “mucosal healing” in the current guidance due to a lack of consensus on its definition and ambiguity in the precise meaning of this term for both clinical trials and daily care. In 2016, the FDA mandated that a claim of mucosal healing should be supported by histological assessment in addition to endoscopy, although the lack of consensus on scoring systems and definitions was acknowledged, leading to the inclusion of this claim in current drug labels.^10^ Furthermore, histological assessment in phase 2 trials was advised to inform effective implementation in phase 3 trials. In the current document, however, “mucosal healing” is abolished and histologic outcomes are considered exploratory. Despite emerging consensus on definitions and implementation in clinical trials,^11^ adequate justification should be provided for histologic endpoint definitions, grading scales, and scoring techniques.

Several minor changes to the draft guidance also deserve mention. A welcome change is that the FDA claims they will consider shorter washout periods than 5 half-lives to reduce patient exposure to corticosteroids as bridging therapy, provided that appropriate close monitoring is in place and that the potential for increased risk of adverse events early in the trial is disclosed in the protocol and informed consent. While the 2016 guidance mandated defining a minimum duration over which a patient is both corticosteroid-free and in remission to define corticosteroid-free remission, the current definition is simplified: patients should be in clinical remission at the end of the trial and without steroid exposure for at least 8–12 weeks.

At present, the impact of these proposed changes on future trials in UC is difficult to predict, as no large trials designed after the publication of the updated guidance have been completed. Nonetheless, even seemingly minor changes could influence comparability between future and past trials. In line with the STRIDE-II consensus, a MES of 0, not ≤ 1, is the therapeutic goal in UC, which underscores the importance of a clear definition.^12^ In the absence of additional clarification from the FDA, “normal appearance of mucosa” suggests that the mucosa should appear as if it had never been affected by disease to qualify for a MES 0. The provision of “inactive disease” in the prior definition, in accordance with the original publication on the Mayo score, allowed for the presence of pseudopolyps, scarring, and mucosal atrophy. The proposed change in definition may result in decreased rates of endoscopic remission in future trials which will not necessarily reflect a decrement in efficacy, but rather an altered definition. At worst, sponsors could adopt conflicting interpretations of the FDA’s definition, hampering comparisons between future trials as well. Ideally, the FDA should adopt a clear position on how to score hallmarks of past disease activity.

The relegation of histologic outcomes to exploratory may prompt sponsors to abandon histological assessment in future trials. Acknowledging the uncertainty of definitions of histologic endpoints, it may well be that targeting histologic remission is superior to targeting symptomatic and endoscopic remission alone. This question is being addressed by the ongoing VERDICT trial (NCT04259138), which is now fully recruited.^13^ Despite the FDA’s current position, trial sponsors should continue to evaluate histologic outcomes, as these data may be helpful to guide treatment decisions in UC in the future and differentiate mechanisms of action.

In our comparison of the guidance documents, we have often cited a lack of empirical evidence to support the FDA’s decision. The fact that a single trial dataset of an investigational agent ultimately shown to be ineffective in UC addressed the concordance between sigmoidoscopy and a full colonoscopy highlights the pressing need for good-quality research in this field. This is an opportunity for cross-trial collaboration to conclusively resolve this issue by conducting full colonoscopies and providing separate endoscopic scores for the distal and proximal colon.

In summary, the 2022 FDA draft guidance for drug development includes recommendations which, for the most part, are not informed by empirical evidence, nor robust explanation of the underlying rationale, which may ultimately complicate the interpretation of future trials and preclude comparisons with past trials. The mandate of colonoscopy remains of concern due to the unnecessary burden and additional risk posed to trial participants.

## Data Availability

No data were generated.
